# Buildings Contemplated and in Progress

**Published:** 1914-07-11

**Authors:** 


### Buildings Contemplated and in Progress.

Ardrossan.—The Ardrossan and the Saltcoats Town Councils are considering a scheme for the extension of the joint hospital at a cost of £1,700.

Birmingham.—The Corporation are considering a scheme for the extension of Holly Moor Lunatic Asylum.

Hull.—The Hull Town Council invite tenders for the erection and drainage of certain buildings forming part of a tuberculosis hospital at the Cottingham Castle estate. Quantities may be obtained from the city architect, Mr. Joseph H. Hirst, Guildhall, Hull.

Letchworth.—The Hitchin Urban District Council invite tenders for the erection of an isolation hospital, with the necessary administration block, laundry, etc. Mr. H. Percy Adams, 28 Woburn Place, Russell Square, W.C., is the architect. Bills of quantities and forms of tender can be obtained upon application to the Clerk to the Council, Mr. A. E. Passingham, 5 Bancroft, Hitchin, Herts.

Melton Mowbray.—A committee has been appointed to develop a scheme for the provision of a cottage hospital.

Motherwell.—The Town Council have decided to enlarge the hospital. The plans for a new pavilion, giving accommodation for twenty-seven additional beds, and estimated to cost £4,500, have been passed by the Dean of Guild Court.

Sheffield.—The Guardians have decided to borrow £16,800 for a children's block at the Union Hospital.

Stockport.—The Guardians have decided to apply to the Local Government Board for sanction to borrow £4,300 for the enlargement of the nurses' home at the Stepping Hill Hospital.

